# Bis(2-bromo­benz­yl) ether

**DOI:** 10.1107/S1600536814011738

**Published:** 2014-06-11

**Authors:** Venkatramu Anuradha, P. Nagendra, S. Madan Kumar, B. P. Siddaraju, N. K. Lokanath

**Affiliations:** a Department of Physics, Dr M. G. R. Educational and Research Institute, Maduravoyal, Chennai, India; b Department of Chemistry, BET Academy of Higher Education, Bharathi College, Bharthi Nagara, Mandya 571 422, India; c Department of Studies in Physics, University of Mysore, Manasagangotri, Mysore 570 006, India; d Department of Chemistry, G. Made Gowda Institute of Technology, Bharthi Nagara, Mandya 571422, India

## Abstract

In the title compound, C_14_H_12_Br_2_O, the dihedral angle between the aromatic rings is 2.7 (3)° and the Br atoms lie on the same side of the mol­ecule. No inter­molecular inter­actions occur in the crystal beyond van der Waals contacts.

## Related literature   

For the use of benzyl groups in organic synthesis, see; Rao & Kumar (2001 ▶); Tareque *et al.* (2006 ▶).
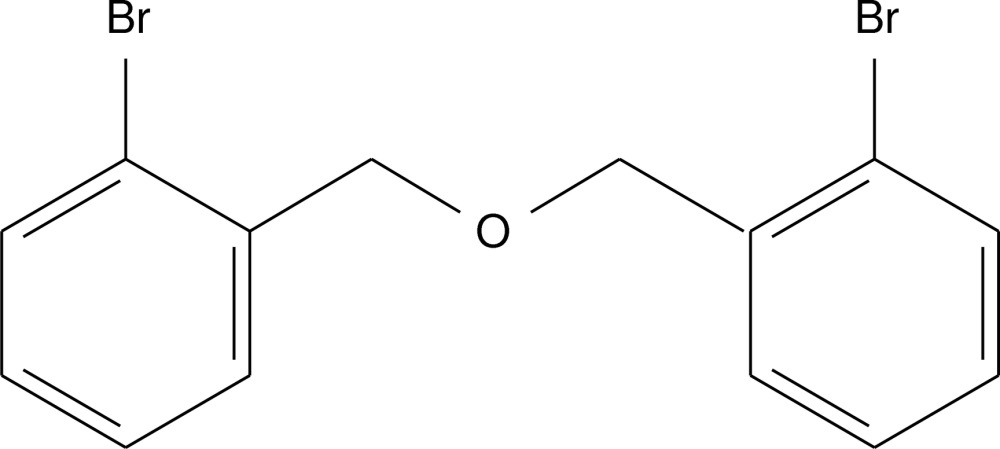


## Experimental   

### 

#### Crystal data  

C_14_H_12_Br_2_O
*M* *_r_* = 356.04
Monoclinic, 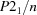

*a* = 11.6022 (6) Å
*b* = 10.1590 (5) Å
*c* = 12.2368 (6) Å
β = 112.853 (2)°
*V* = 1329.10 (12) Å^3^
*Z* = 4
Cu *K*α radiation
μ = 7.58 mm^−1^
*T* = 296 K
0.23 × 0.22 × 0.21 mm

#### Data collection  

Bruker X8 Proteum diffractometer
Absorption correction: multi-scan (*SADABS*; Bruker, 2013) *T*_min_ = 0.275, *T*_max_ = 0.299
10361 measured reflections
2185 independent reflections
1957 reflections with *I* > 2σ(*I*)
*R*_int_ = 0.054

#### Refinement  

*R*[*F* ^2^ > 2σ(*F* ^2^)] = 0.073
*wR*(*F* ^2^) = 0.192
*S* = 1.07
2185 reflections
155 parameters
H-atom parameters constrained
Δρ_max_ = 1.26 e Å^−3^
Δρ_min_ = −1.61 e Å^−3^

### 

Data collection: *APEX2* (Bruker, 2013 ▶); cell refinement: *SAINT* (Bruker, 2013 ▶); data reduction: *SAINT*; program(s) used to solve structure: *SHELXS97* (Sheldrick, 2008 ▶); program(s) used to refine structure: *SHELXL97* (Sheldrick, 2008 ▶); molecular graphics: *Mercury* (Macrae *et al.*, 2008 ▶); software used to prepare material for publication: *Mercury*.

## Supplementary Material

Crystal structure: contains datablock(s) global, I. DOI: 10.1107/S1600536814011738/hb7230sup1.cif

Structure factors: contains datablock(s) I. DOI: 10.1107/S1600536814011738/hb7230Isup2.hkl

Supporting information file. DOI: 10.1107/S1600536814011738/hb7230Isup3.cml

CCDC reference: 1004401

Additional supporting information:  crystallographic information; 3D view; checkCIF report

## Figures and Tables

**Fig. 1.**
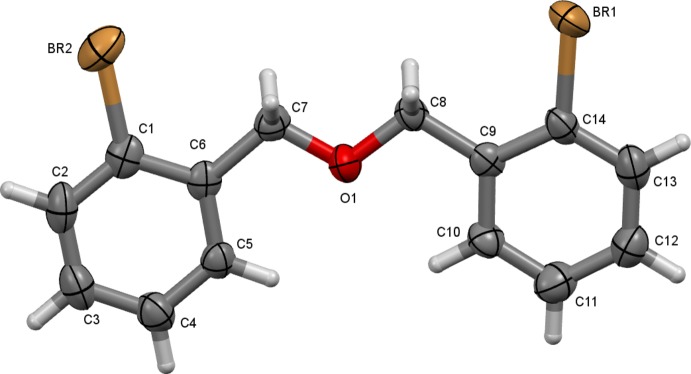
A view of the title molecule, with displacement ellipsoids drawn at the 50% probability level.

**Fig. 2.**
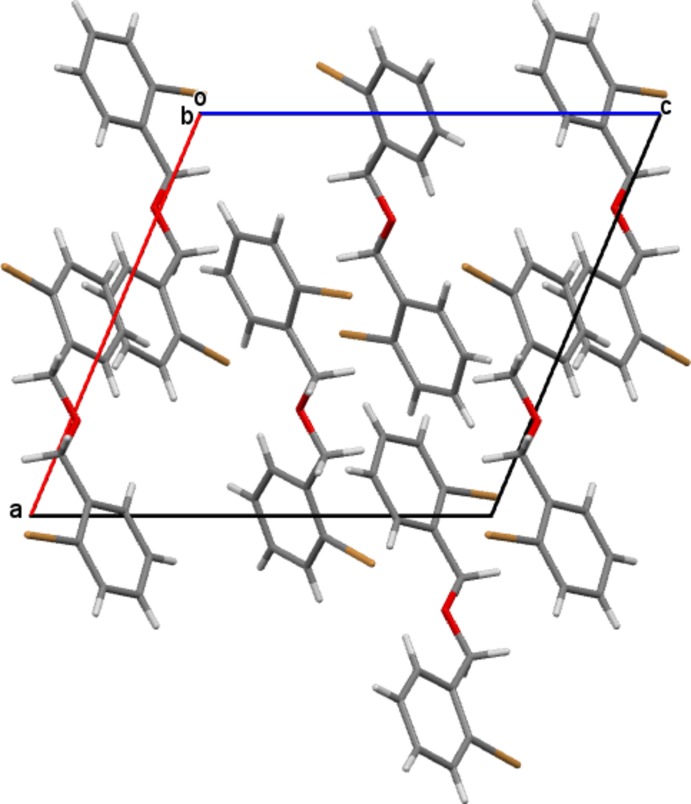
A viewed along the b axis of the crystal packing of the title compound.

## supplementary crystallographic information

### S1. Comment

Benzyl groups are commonly used for the protection of alcohol and phenol moieties for synthesis. The benzyl alcohol used in the benzylation of phenol (Tareque, *et al.*,, 2006). The benzyl ethers are used as intermediates in sigmatropic rearrangement reactions such as Claisen and the Cope rearrangements (Rao and Kumar, 2001).

In the title compound, C_14_H_10_Br_2_O, (Fig. 1), the dihedral angle between the aromatic rings is 2.7 (3)° and the Br atoms lie on the same side of the molecule. No intermolecular interactions occur in the crystal beyond van der Waals' contacts.

### S2. Experimental

2-Bromobenzyl alcohol (1.87 g, 0.01 mol), sodium hydride 0.24 g, 0.01 mol) and 2-bromobenzyl bromide (2.52 g, 0.01 mol) were ground well and mixed in 25 ml of THF. The mixture were stirred in a beaker at 60 °C for one hour. The mixture was kept aside for five days at room temperature in a vaccum desiccator over phosphorous pentoxide. The colourless crystals were obtained by slow evaporation (*M*. P. 374 - 376 K). Colourless blocks were obtained from slow evaporation of a solution of ethylacetate.

### S3. Refinement

The hydrogen atom were fixed geometrically (C—H=0.93–0.96 Å) and allowed to ride on their parent atoms with *U*_iso_(H) = 1.2*U*_eq_(C).

### Crystal data

**Table:**

C_14_H_12_Br_2_O	*F*(000) = 688
*M**_r_* = 356.04	*D*_x_ = 1.769 Mg m^−^^3^
Monoclinic, *P*2_1_/*n*	Cu *K*α radiation, λ = 1.54178 Å
Hall symbol: -P 2yn	Cell parameters from 2185 reflections
*a* = 11.6022 (6) Å	θ = 4.5–64.7°
*b* = 10.1590 (5) Å	µ = 7.58 mm^−^^1^
*c* = 12.2368 (6) Å	*T* = 296 K
β = 112.853 (2)°	Block, colourless
*V* = 1329.10 (12) Å^3^	0.23 × 0.22 × 0.21 mm
*Z* = 4	

### Data collection

**Table:**

Bruker X8 Proteum diffractometer	2185 independent reflections
Radiation source: Bruker MicroStar microfocus rotating anode	1957 reflections with *I* > 2σ(*I*)
Helios multilayer optics monochromator	*R*_int_ = 0.054
Detector resolution: 10.7 pixels mm^-1^	θ_max_ = 64.7°, θ_min_ = 4.5°
φ and ω scans	*h* = −5→13
Absorption correction: multi-scan (*SADABS*; Bruker, 2013)	*k* = −11→11
*T*_min_ = 0.275, *T*_max_ = 0.299	*l* = −14→11
10361 measured reflections	

### Refinement

**Table:**

Refinement on *F*^2^	Secondary atom site location: difference Fourier map
Least-squares matrix: full	Hydrogen site location: inferred from neighbouring sites
*R*[*F*^2^ > 2σ(*F*^2^)] = 0.073	H-atom parameters constrained
*wR*(*F*^2^) = 0.192	*w* = 1/[σ^2^(*F*_o_^2^) + (0.137*P*)^2^ + 1.9645*P*] where *P* = (*F*_o_^2^ + 2*F*_c_^2^)/3
*S* = 1.07	(Δ/σ)_max_ < 0.001
2185 reflections	Δρ_max_ = 1.26 e Å^−^^3^
155 parameters	Δρ_min_ = −1.61 e Å^−^^3^
0 restraints	Extinction correction: *SHELXL97* (Sheldrick, 2008), FC^*^=KFC[1+0.001XFC^2^Λ^3^/SIN(2Θ)]^-1/4^
Primary atom site location: structure-invariant direct methods	Extinction coefficient: 0.0219 (17)

### Special details

**Table:**

Geometry. Bond distances, angles etc. have been calculated using the rounded fractional coordinates. All su's are estimated from the variances of the (full) variance-covariance matrix. The cell esds are taken into account in the estimation of distances, angles and torsion angles
Refinement. Refinement on F^2^ for ALL reflections except those flagged by the user for potential systematic errors. Weighted R-factors wR and all goodnesses of fit S are based on F^2^, conventional R-factors R are based on F, with F set to zero for negative F^2^. The observed criterion of F^2^ > 2sigma(F^2^) is used only for calculating -R-factor-obs etc. and is not relevant to the choice of reflections for refinement. R-factors based on F^2^ are statistically about twice as large as those based on F, and R-factors based on ALL data will be even larger.

### Fractional atomic coordinates and isotropic or equivalent isotropic displacement parameters (Å^2^)

**Table:**

	*x*	*y*	*z*	*U*_iso_*/*U*_eq_	
Br1	0.45265 (7)	0.18786 (6)	0.48911 (7)	0.0610 (3)	
Br2	1.11871 (7)	0.50492 (7)	0.78422 (6)	0.0629 (4)	
O1	0.7350 (4)	0.4954 (4)	0.4857 (4)	0.0441 (14)	
C1	1.0514 (5)	0.6241 (6)	0.6552 (5)	0.0410 (17)	
C2	1.1258 (5)	0.7260 (7)	0.6461 (6)	0.051 (2)	
C3	1.0785 (6)	0.8126 (6)	0.5532 (6)	0.052 (2)	
C4	0.9584 (6)	0.7975 (6)	0.4701 (6)	0.0488 (19)	
C5	0.8845 (5)	0.6954 (5)	0.4814 (5)	0.0391 (17)	
C6	0.9296 (5)	0.6058 (5)	0.5729 (5)	0.0348 (16)	
C7	0.8506 (5)	0.4919 (5)	0.5835 (5)	0.0389 (17)	
C8	0.6547 (5)	0.3941 (5)	0.4916 (5)	0.0384 (16)	
C9	0.5324 (5)	0.4041 (5)	0.3857 (5)	0.0350 (16)	
C10	0.5112 (5)	0.5001 (5)	0.3003 (5)	0.0411 (17)	
C11	0.3985 (6)	0.5106 (7)	0.2048 (6)	0.053 (2)	
C12	0.3028 (6)	0.4217 (6)	0.1926 (5)	0.0499 (17)	
C13	0.3212 (6)	0.3256 (6)	0.2769 (6)	0.0493 (19)	
C14	0.4339 (5)	0.3178 (5)	0.3721 (5)	0.0401 (16)	
H2	1.20690	0.73590	0.70220	0.0610*	
H3	1.12770	0.88170	0.54620	0.0630*	
H4	0.92700	0.85570	0.40670	0.0590*	
H5	0.80290	0.68710	0.42610	0.0460*	
H7A	0.89290	0.40930	0.58440	0.0470*	
H7B	0.83730	0.49890	0.65680	0.0470*	
H8A	0.64000	0.40210	0.56410	0.0460*	
H8B	0.69290	0.30910	0.49190	0.0460*	
H10	0.57470	0.55950	0.30740	0.0490*	
H11	0.38640	0.57660	0.14880	0.0630*	
H12	0.22690	0.42740	0.12790	0.0600*	
H13	0.25770	0.26610	0.26950	0.0590*	

### Atomic displacement parameters (Å^2^)

**Table:**

	*U*^11^	*U*^22^	*U*^33^	*U*^12^	*U*^13^	*U*^23^
Br1	0.0563 (6)	0.0420 (5)	0.0818 (7)	−0.0090 (3)	0.0236 (4)	0.0184 (3)
Br2	0.0562 (6)	0.0755 (7)	0.0436 (6)	0.0143 (3)	0.0047 (4)	−0.0006 (3)
O1	0.031 (2)	0.047 (2)	0.054 (3)	−0.0052 (16)	0.0161 (18)	0.0079 (16)
C1	0.039 (3)	0.048 (3)	0.038 (3)	0.004 (2)	0.017 (2)	−0.014 (2)
C2	0.035 (3)	0.058 (4)	0.058 (4)	−0.009 (3)	0.015 (3)	−0.025 (3)
C3	0.046 (3)	0.047 (4)	0.068 (4)	−0.014 (3)	0.028 (3)	−0.016 (3)
C4	0.048 (3)	0.039 (3)	0.061 (4)	−0.003 (2)	0.023 (3)	−0.004 (3)
C5	0.033 (3)	0.037 (3)	0.046 (3)	0.002 (2)	0.014 (2)	−0.004 (2)
C6	0.031 (2)	0.037 (3)	0.041 (3)	0.0037 (19)	0.019 (2)	−0.011 (2)
C7	0.034 (3)	0.043 (3)	0.041 (3)	0.003 (2)	0.016 (2)	0.002 (2)
C8	0.030 (2)	0.037 (3)	0.052 (3)	−0.001 (2)	0.020 (2)	0.007 (2)
C9	0.033 (2)	0.033 (3)	0.046 (3)	0.004 (2)	0.023 (2)	−0.002 (2)
C10	0.042 (3)	0.043 (3)	0.044 (3)	−0.003 (2)	0.023 (3)	0.005 (2)
C11	0.048 (4)	0.062 (4)	0.052 (4)	0.008 (3)	0.023 (3)	0.011 (3)
C12	0.040 (3)	0.056 (3)	0.049 (3)	0.003 (3)	0.012 (2)	−0.002 (3)
C13	0.039 (3)	0.045 (3)	0.064 (4)	−0.008 (2)	0.020 (3)	−0.013 (3)
C14	0.037 (3)	0.029 (2)	0.060 (3)	−0.0028 (19)	0.025 (3)	−0.004 (2)

### Geometric parameters (Å, º)

**Table:**

Br1—C14	1.897 (5)	C11—C12	1.394 (10)
Br2—C1	1.900 (6)	C12—C13	1.375 (9)
O1—C7	1.409 (8)	C13—C14	1.375 (9)
O1—C8	1.409 (7)	C2—H2	0.9300
C1—C2	1.380 (9)	C3—H3	0.9300
C1—C6	1.393 (8)	C4—H4	0.9300
C2—C3	1.372 (9)	C5—H5	0.9300
C3—C4	1.378 (10)	C7—H7A	0.9700
C4—C5	1.386 (9)	C7—H7B	0.9700
C5—C6	1.379 (8)	C8—H8A	0.9700
C6—C7	1.513 (8)	C8—H8B	0.9700
C8—C9	1.508 (8)	C10—H10	0.9300
C9—C10	1.380 (8)	C11—H11	0.9300
C9—C14	1.398 (8)	C12—H12	0.9300
C10—C11	1.378 (9)	C13—H13	0.9300
			
C7—O1—C8	111.6 (4)	C2—C3—H3	120.00
Br2—C1—C2	118.5 (5)	C4—C3—H3	120.00
Br2—C1—C6	119.3 (4)	C3—C4—H4	120.00
C2—C1—C6	122.2 (6)	C5—C4—H4	120.00
C1—C2—C3	119.1 (6)	C4—C5—H5	119.00
C2—C3—C4	120.3 (6)	C6—C5—H5	119.00
C3—C4—C5	119.8 (6)	O1—C7—H7A	110.00
C4—C5—C6	121.4 (6)	O1—C7—H7B	110.00
C1—C6—C5	117.2 (5)	C6—C7—H7A	110.00
C1—C6—C7	121.2 (5)	C6—C7—H7B	110.00
C5—C6—C7	121.5 (5)	H7A—C7—H7B	108.00
O1—C7—C6	108.5 (4)	O1—C8—H8A	110.00
O1—C8—C9	109.1 (4)	O1—C8—H8B	110.00
C8—C9—C10	122.0 (5)	C9—C8—H8A	110.00
C8—C9—C14	120.9 (5)	C9—C8—H8B	110.00
C10—C9—C14	117.1 (5)	H8A—C8—H8B	108.00
C9—C10—C11	121.9 (6)	C9—C10—H10	119.00
C10—C11—C12	119.7 (6)	C11—C10—H10	119.00
C11—C12—C13	119.6 (6)	C10—C11—H11	120.00
C12—C13—C14	119.7 (6)	C12—C11—H11	120.00
Br1—C14—C9	119.9 (4)	C11—C12—H12	120.00
Br1—C14—C13	118.2 (5)	C13—C12—H12	120.00
C9—C14—C13	122.0 (5)	C12—C13—H13	120.00
C1—C2—H2	120.00	C14—C13—H13	120.00
C3—C2—H2	120.00		
			
C8—O1—C7—C6	−178.2 (5)	C5—C6—C7—O1	2.3 (7)
C7—O1—C8—C9	179.3 (5)	O1—C8—C9—C10	0.5 (7)
Br2—C1—C2—C3	179.8 (5)	O1—C8—C9—C14	−177.6 (5)
C6—C1—C2—C3	0.2 (10)	C8—C9—C10—C11	−178.7 (6)
Br2—C1—C6—C5	179.5 (4)	C14—C9—C10—C11	−0.6 (9)
Br2—C1—C6—C7	−0.8 (8)	C8—C9—C14—Br1	1.0 (7)
C2—C1—C6—C5	−0.9 (9)	C8—C9—C14—C13	179.5 (6)
C2—C1—C6—C7	178.9 (6)	C10—C9—C14—Br1	−177.2 (4)
C1—C2—C3—C4	−0.2 (10)	C10—C9—C14—C13	1.3 (9)
C2—C3—C4—C5	0.9 (10)	C9—C10—C11—C12	−0.5 (10)
C3—C4—C5—C6	−1.7 (10)	C10—C11—C12—C13	0.9 (10)
C4—C5—C6—C1	1.6 (9)	C11—C12—C13—C14	−0.3 (10)
C4—C5—C6—C7	−178.2 (6)	C12—C13—C14—Br1	177.7 (5)
C1—C6—C7—O1	−177.4 (5)	C12—C13—C14—C9	−0.9 (10)

### Hydrogen-bond geometry (Å, º)

**Table:**

*D*—H···*A*	*D*—H	H···*A*	*D*···*A*	*D*—H···*A*
C5—H5···O1	0.93	2.32	2.685 (7)	103
C10—H10···O1	0.93	2.34	2.705 (8)	103

## References

[bb1] Bruker (2013). *APEX2*, *SAINT* and *SADABS* Bruker AXS Inc., Madison,Wisconsin, USA.

[bb2] Macrae, C. F., Bruno, I. J., Chisholm, J. A., Edgington, P. R., McCabe, P., Pidcock, E., Rodriguez-Monge, L., Taylor, R., van de Streek, J. & Wood, P. A. (2008). *J. Appl. Cryst.* **41**, 466–470.

[bb3] Rao, H. S. P. & Kumar, S. S. P. (2001). *Proc. Indian Acad. Sci. (Chem. Sci.)*, **113**, 191–196.

[bb4] Sheldrick, G. M. (2008). *Acta Cryst.* A**64**, 112–122. 10.1107/S0108767307043930 18156677

[bb5] Tareque, M. H., Ismail, M., Chakravarthy, P. & Rana, A. A. R. (2006). *Banglad. J. Sci. Ind. Res.* **41**, 257–261.

